# Automated staging of zebrafish embryos using machine learning

**DOI:** 10.12688/wellcomeopenres.18313.3

**Published:** 2023-04-26

**Authors:** Rebecca A. Jones, Matthew J. Renshaw, David J. Barry, James C. Smith

**Affiliations:** 1 Developmental Biology Laboratory, The Francis Crick Institute, 1 Midland Road, London, NW1 1AT, UK; 2 Department of Molecular Biology, Princeton University, Princeton, NJ, 08544, USA; 3 Crick Advanced Light Microscopy (CALM), The Francis Crick Institute, 1 Midland Road, London, NW1 1AT, UK

**Keywords:** Zebrafish, development, machine learning, staging, developmental delay, classifier

## Abstract

The zebrafish ( *Danio rerio*), is an important biomedical model organism used in many disciplines, including development, disease modeling and toxicology, to better understand vertebrate biology. The phenomenon of developmental delay in zebrafish embryos has been widely reported as part of a mutant or treatment-induced phenotype, and accurate characterization of such delays is imperative. Despite this, the only way at present to identify and quantify these delays is through manual observation, which is both time-consuming and subjective. Machine learning approaches in biology are rapidly becoming part of the toolkit used by researchers to address complex questions. In this work, we introduce a machine learning-based classifier that has been trained to detect temporal developmental differences across groups of zebrafish embryos. Our classifier is capable of rapidly analyzing thousands of images, allowing comparisons of developmental temporal rates to be assessed across and between experimental groups of embryos. Finally, as our classifier uses images obtained from a standard live-imaging widefield microscope and camera set-up, we envisage it will be readily accessible to the zebrafish community, and prove to be a valuable resource.

## Introduction

The zebrafish ( *Danio rerio*) is a model organism widely used in a variety of fields, including developmental biology, disease modelling, cancer biology and immunology ( Choi *et al.,* 2021; Eisen, 1996; Gomes & Mostowy, 2020; Kemmler *et al.,* 2021; Zanandrea *et al.,* 2020). External fertilization, high fecundity, low cost and ease of genetic manipulation together make zebrafish a valuable model for many studies, and their transparent embryos make them particularly useful in studies of developmental biology ( Nüsslein-Volhard, 2012). The advent of CRISPR/Cas (Clustered Regularly Interspaced Short Palindromic Repeats/CRISPR associated (protein)) technology has meant that many studies use transgenic lines to answer important biological questions ( Liu *et al.,* 2019).

Zebrafish embryos develop externally, becoming free-swimming, independently feeding larvae by five days post fertilization (dpf) ( Kimmel *et al.,* 1995). Development is rapid, with gastrulation and neurulation occurring within the first 12 hours post fertilization (hpf) ( Kimmel *et al.,* 1995). Many studies, and particularly developmental studies, require accurate staging of zebrafish embryos and larvae. Although the timing of fertilization can be estimated to within ~30 minutes, the numbers of hours post fertilization at the standard temperature of 28.5°C ( Kimmel *et al.,* 1995) provides only an approximation of the actual developmental stage, because other factors, like population density and water quality, can affect maturation rates ( Singleman & Holtzman, 2014). Even when such factors are controlled for, embryos within a clutch may develop at different rates ( Parichy *et al.,* 2009). Researchers therefore use both hpf/dpf and staging guides that are based on morphological criteria to stage individual embryos ( Kimmel *et al.,* 1995). These morphological features include the number of somites and the appearance of landmark structures such as the embryonic shield, tail bud and eye primordium ( Kimmel *et al.,* 1995; Westerfield, 2000).

Staging of embryos is of particular importance because many studies report ‘developmental delay’ as part of a genetic or drug-induced phenotype. For example, transgenic lines might develop more slowly than their wild-type (WT) counterparts, ( Elabd *et al.,* 2019; Giraldez *et al.,* 2005; Jia *et al.,* 2020; Li *et al.,* 2017), as might embryos injected with antisense morpholino oligonucleotides ( Flinn *et al.,* 2008; Hung *et al.,* 2013; Walpita *et al.,* 2010), or those treated with drugs ( Akthar *et al.,* 2019; Byrnes *et al.,* 2018; Farooq *et al.,* 2019). Significantly, zebrafish have emerged as important models in which to study the effects of environmental and aquatic toxins, with many of these treatments also resulting in a developmental delay ( Aksakal & Sisman, 2020; Li *et al.,* 2020; Mesquita *et al.,* 2017). Such delays are difficult to quantify without manually staging large numbers of embryos, which is inconvenient, subjective and time-consuming, especially when assessing developmental abnormalities ( Jeanray *et al.,* 2015; Teixidó *et al.,* 2019). Adding to this difficulty, the delay is often temporary, and transgenic or treated embryos ‘catch up’ with their WT counterparts ( Elabd *et al.,* 2019; Ge *et al.,* 2019; Kamei *et al.,* 2018). It is therefore important to identify exactly when the delay is occurring to account for it in the study. Conversely, in many studies it is necessary to exclude general developmental delay, a potentially confounding variable, as the cause of either a tissue-specific phenotype or a developmental delay induced by a drug treatment or specific mutation, in order to validate the results of a given experiment ( Mannucci *et al.,* 2021; Sidik *et al.,* 2021). For example, if one knocks out a gene involved in cardiac development, it is important to determine if any delay in heart formation is cardiac-specific, or part of an organism-wide developmental delay. In some studies, altered hatching rates are used as an additional proxy for developmental stage ( Martinez *et al.,* 2018; Tshering *et al.,* 2021; Zhang *et al.,* 2015), yet hatching defects can be caused by hatching gland specific issues, as opposed to a more general developmental delay ( Suzuki *et al.,* 2019; Trikić *et al.,* 2011). Because assessing developmental delay is such a critical part of zebrafish related work, it is imperative that we develop a more standardized and automated way to measure it: one that reduces the time and subjectivity burden inherent in manual staging.

The use of image analysis has become increasingly popular in the life sciences, automating the quantification of microscopy images in an unbiased fashion ( Meijering *et al.,* 2016). However, designing an image analysis algorithm to detect the wide range of morphological features on which staging guides depend would be a challenging endeavor. Nevertheless, the staging of embryos based on microscopy images is a task to which machine learning is well-suited. Machine learning approaches, where a computer program uses algorithms and statistical models to continuously learn and improve pattern prediction, is already used widely in biological studies ( Greener *et al.,* 2022). Several labs have already made successful attempts to automate the analysis of morphological features of zebrafish embryos using machine learning. Jeanray *et al.* (2015) used a supervised machine learning approach to classify bright-field images of zebrafish embryos according to chemical treatment induced defects, with >90% concordance to manual expert classification, and various other studies have produced similar classifiers ( Ishaq *et al.,* 2017; Shang *et al.,* 2020). More recently, Guglielmi *et al.* (2021) used an innovative optical projection tomography (OPT) and back-projection technique followed by semi-automated segmentation and quantitation to objectively describe the morphological features of zebrafish embryos in which BMP signaling was perturbed. In terms of developmental staging, Pond *et al.* (2021) recently developed a convolutional neural network (CNN)-based classifier to stage zebrafish tail-buds at four discrete developmental stages, demonstrating that high accuracy can be achieved with small data sets (<100 images). These elegant systems highlight the power of machine learning approaches in the identification of morphological features and discrete developmental stages, but none of these studies extract sufficient information to enable complete temporal developmental profiles to be compared. For example, Pond *et al.* (2021) compared four developmental stages, and whilst their CNN-based classifier was able to accurately predict these stages, this is not sufficient to extract a comparable developmental profile.

Using a combination of live imaging and machine learning approaches, we have developed a classifier to quantify zebrafish embryonic development, allowing objective and meaningful relative comparisons over time. Moreover, we demonstrate our classifier's ability to stage specific developmental time-points is comparable to human experts. This work provides proof of principle that machine learning algorithms can be used to accurately stage zebrafish embryos and we hope that our classifier will become a valuable resource for the zebrafish community.

## Methods

### Zebrafish husbandry

All zebrafish work, including housing and husbandry, was undertaken in accordance with institutional (The Francis Crick Institute) and national (UK) ethical and animal welfare regulations, including the Crick Use of Animals in Research Policy, the Animals (Scientific Procedures) Act 1986 (ASPA) implemented by the Home Office in the UK and the Animal Welfare Act 2006. All regulated procedures were carried out at The Francis Crick Institute in accordance with UK Home Office regulations under project license PF59163DB, which underwent full ethical review and approval by The Francis Crick Institute’s Animal Ethics Committee. Consideration was given to the ‘3Rs’ in experimental design, and animals were observed on a daily basis for any signs of illness/distress. Any animals displaying evidence of suffering (physiological/behavioral changes, signs of injury) were euthanized in pH neutralized MS222 for a minimum of 30 minutes, before a second physical euthanasia method was performed. The Zirc AB line was used in all experiments. For most experiments, zebrafish embryos were obtained by tank mass-spawning using either a mating tank with a clear Perspex divider or a Mass Embryo Production (MEP) system (MBK Installations). Embryos were collected 30 minutes following divider removal or first-light respectively, and then at 30-minute intervals thereafter until spawning ceased. Embryos were maintained in plates of ~50 animals, at 28.5°C in E2 medium, prepared by The Francis Crick Institute’s Media Preparation Facility. Approximately 30 minutes prior to imaging, zebrafish embryos were manually checked for correct development, then individually transferred into separate wells of a 96-well plate, containing pre-warmed (28.5°C or 25°C) E2 medium. One plate of 96 embryos was then transferred to the Crick Advanced Light Microscopy (CALM) Science Technology Platform (STP) imaging suite and mounted in the environmental chamber (see below). One 96-well plate was used for imaging each condition, as a 96-well plate set-up provided optimal conditions for individual embryo image capture. Having 96 embryos per condition also ensured that if several embryos failed to develop normally, there would still be sufficient embryos to perform both training and downstream analysis. Excess embryos were disposed of in MS222 as above.

### Live imaging

**Figure 1.**
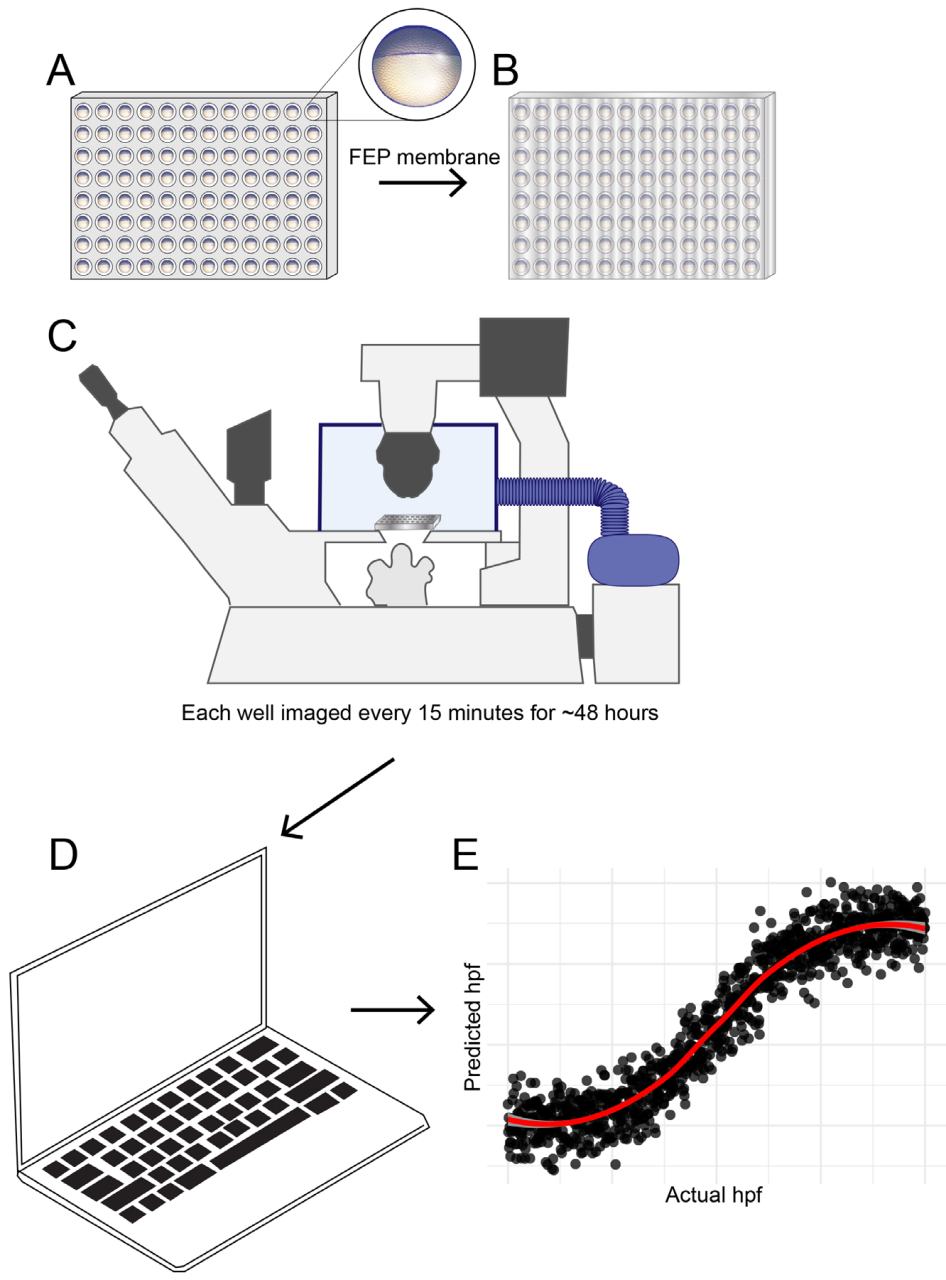
Schematic diagram showing developmental temporal quantification workflow. Zebrafish embryos were individually seeded into U-bottomed 96 well plates in E2 medium at around 3.5 hpf. ( **A**) The plate was then sealed with a breathable FEP membrane ( **B**) and transferred to an inverted microscope with motorised XY stage where the temperature was maintained at 25.0 or 28.5°C using an environmental chamber ( **C**). Brightfield images were captured of each well every 15 mins from sphere stage (4hpf) until 18 hpf. Images were analysed using an ilastik object classification pipeline ( **D**) to produce plots showing predicted hpf versus actual hpf, similar to the schematic plot shown ( **E**).

Zebrafish embryos were maintained at 28.5°C until shortly before four hpf as defined by both hpf and morphological criteria (sphere stage, ( Kimmel *et al.,* 1995)), at which point they were transferred into U-bottomed 96-well plates (Thermo Fisher) in E2 medium as described above. Plates were covered with fluorinated ethylene propylene (FEP) membrane (1 mil Teflon FEP film, American Durafilm) to prevent condensation and allow for gas exchange. Brightfield images of embryos individually seeded in 96-well plates were acquired every 15 minutes starting at four hpf for 60 hours using a Nikon Ti2 microscope with 2X/0.1 Plan Apo objective and 1.5x intermediate magnification. A small pixel complementary metal-oxide-semiconductor (CMOS) camera (UI-3280SE, iDS) enabled a whole embryo to be captured in a single field of view at cellular resolution (pixel size 1.15 μm). Sample temperature was maintained at either 25.0 or 28.5°C using an environmental chamber enclosure (Okolab). The microscope was controlled with Micro-Manager v2.0 software ( Edelstein *et al.,* 2014, RRID:SCR_000415) and the HCS Site Generator plugin was used to generate a list of positions for the 96-well plate. The workflow is summarized in Figure 1.

### Machine learning pipeline configuration

Automated staging of zebrafish embryos was performed using ilastik ( Berg *et al.,* 2019) and FIJI ( Schindelin *et al.,* 2012), both free, open source software popular among life science researchers. All the FIJI scripts and ilastik project files needed to reproduce these steps are available to download online ( Barry, 2022). This repository contains step-by-step instructions that can be used to either reproduce the raw data used to generate the plots in this manuscript, or run the classifier on new data (see README.md in Barry, 2022).

### Training of machine learning model

**Figure 2.**
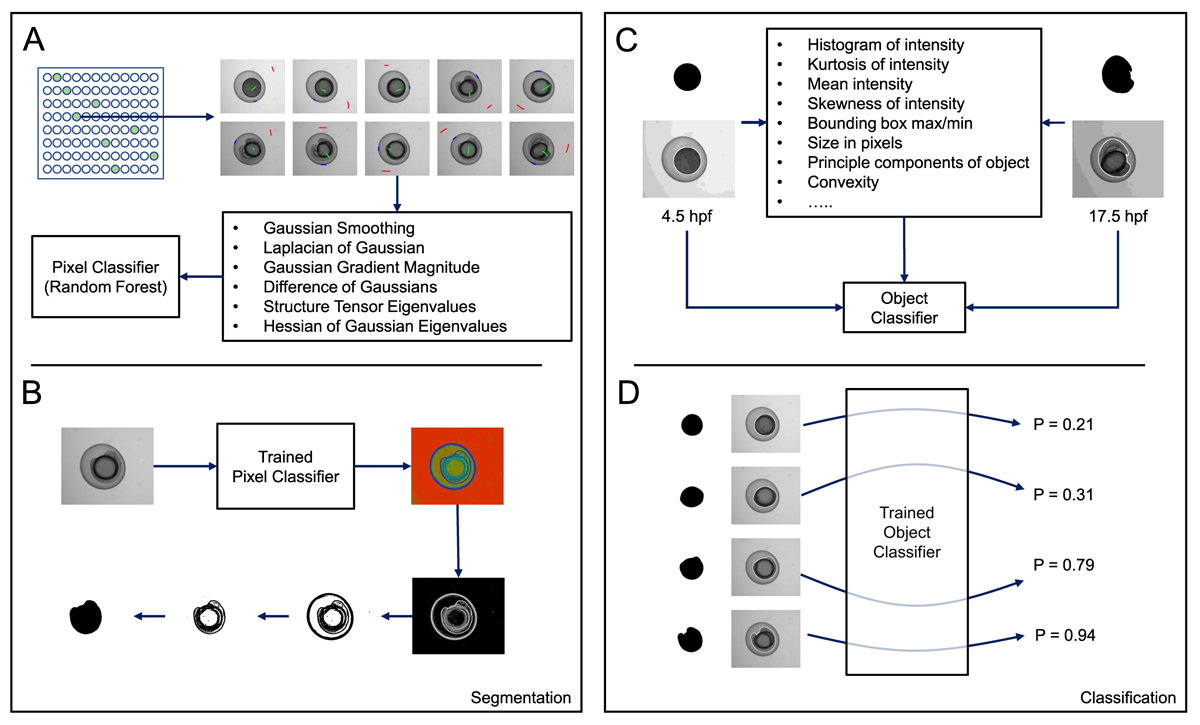
Overview of ilastik-based pixel and object classification pipeline. ( **A**) A pixel classifier was trained to segment the embryos in each image. Using a random selection of time-points, regions in images were manually annotated as either background (red), embryo (green), or boundary (blue). Using the measures shown, calculated at various different scales, and the annotations, ilastik then trained a random-forest classifier. ( **B**) When supplied with test data, the trained pixel classifier produced three probability maps, one for each of the classes listed in ( **A**) (background, embryo, boundary). Each pixel in a map for a particular class gives the probability that the pixel belongs to that particular class. By thresholding the boundary probability map and performing some simple morphological processing on the resulting binary image, we could obtain a mask representing the embryo. ( **C**) We then trained an object classification pipeline using ilastik. Using the mask images generated in ( **B**) and the corresponding raw images, an object classifier was trained to recognise either 4.5 or 17.5 hpf embryos. ( **D**) When supplied with test mask and raw images, the trained object classifier returned a probability corresponding to the likelihood that the test image represented a 4.5 (P = 0.0) or 17.5 hpf (P = 1.0) embryo.

Images of zebrafish embryos were randomly divided into training and test datasets as described in Table 1. Using an ilastik pixel classification pipeline, pixels in training data were manually labelled as belonging to one of three classes: embryo, background or embryo/background boundary ( Figure 2A). These labels, together with a range of generic pixel features, were used to then train a random forest classifier using ilastik’s pixel classification workflow. All training labels and pixel features used to train the pixel classifier can be viewed in the ilastik pixel classifier project file (PixelClassifier.ilp - Barry, 2022).

**Table 1.** Overview of datasets used for training and testing.

Plate	Incubation Temperature (°C)	Wells Used for Training	Wells Used for Testing
1	28.5	13	82
2	28.5	11	84
3	25.0	0	95

The probability map for the boundary class output by ilastik was then used to fully segment the embryos, using simple grey level thresholding in FIJI ( Figure 2B; see Segment.ijm - Barry, 2022). The resultant embryo masks were then combined with the corresponding raw embryo images to train an ilastik object classification pipeline (ObjectClassifier.ilp; Barry, 2022). In such a pipeline, ilastik calculates various morphological features based on the connected components in the masks and a series of intensity features drawn from the pixel values in the raw images within the regions delineated by the masks (see https://www.ilastik.org/documentation/objects/objects for further information). We manually labelled 4.5 and 17.5 hpf embryos in our training data ( Table 1) and trained the ilastik object classifier based on these annotations ( Figure 2C). All training labels and pixel features used to train the object classifier can be viewed in the ilastik object classifier project file (ObjectClassifier.ilp – Barry, 2022).

The trained object classifier, when challenged with new test data, outputs two values; one gives the probability that the embryo is 4.5 hpf ( *p _4.5_*), the other that the embryo is 17.5 hpf ( *p _17.5_*). The sum of these two probabilities is always 1.0. Given the probability of a given test embryo being 17.5 hpf, the predicted hpf is calculated as follows:

$$P_{hpf}\;\text{=}\;\text{4.5}\;\text{+}\;p_{\text{17.5}}\;\text{×}\;\text{(17.5}\;\text{–}\;\text{4.5)}$$

### Comparison with manual (human) staging

To enable comparisons to be made between the accuracy of the classifier and manual (human) staging of zebrafish embryos, three individuals were asked to stage WT zebrafish embryos in 42 still images, randomly selected from the time-lapse movies that the classifier had previously analyzed. All were provided with the standard staging guide of Kimmel *et al.* (1995). These data were then compared with the predicted hpf generated by our classifier, for the same 42 images. The range of error was then calculated as the difference between the maximum and minimum error.

### Statistical analysis

**Figure 3.**
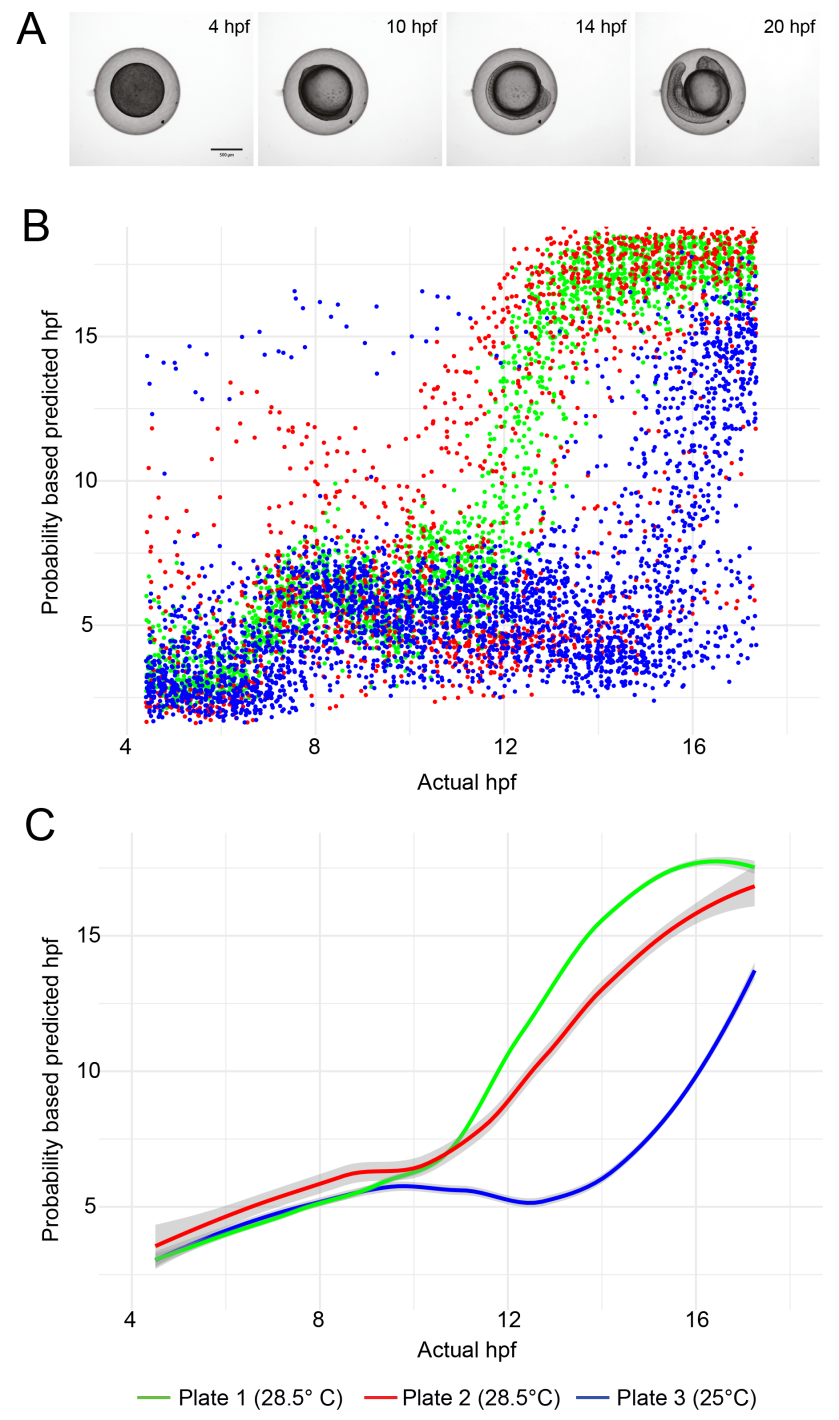
Temporal development profile of zebrafish embryos. ( **A**) Examples of still images from time-lapse movies used to train the object classification algorithm. ( **B**) Scatter plot showing the hpf predicted by the ilastik object classifier versus the actual hpf for each embryo image. Each dataset contains approximately 5,000 data points (96 wells per experiment, imaged every 15 minutes for 13 hours). ( **C**) Line fit of the data in ( **B**) using locally estimated scatterplot smoothing (LOESS). The grey region around each line shows the 95% confidence interval.

LOESS (locally established scatter plot smoothing) was used to generate the line plots in Figure 3 showing the temporal development profile of embryos maintained at 28.5°C compared to 25°C. 95% confidence intervals (CI) calculated in R are displayed. All R scripts are available in the software availability section ( Barry, 2022).

## Results

Development of embryos is slower when they are maintained at 25°C than at 28.5°C. As proof of principle, we challenged our trained machine learning model with previously unseen test data, consisting of embryos incubated at different temperatures ( Table 1). The classifier was able to clearly differentiate between embryos maintained at 28.5°C and those maintained at 25°C ( Figure 3). For the test data derived from plates incubated at 28.5°C, a greater spread in datapoints is evident for data derived from one plate versus the other ( Figure 3C). The standard error of the mean predicted hpf, averaged over all timepoints, was 0.16 hpf for Plate 1 versus 0.60 hpf for Plate 2. This may be because slightly more training data was drawn from plate 1. But it should also be considered that the test data was not subjected to any quality control, so it is possible that more embryos on plate 2 died or drifted out of the field of view than on plate 1.

**Figure 4.**
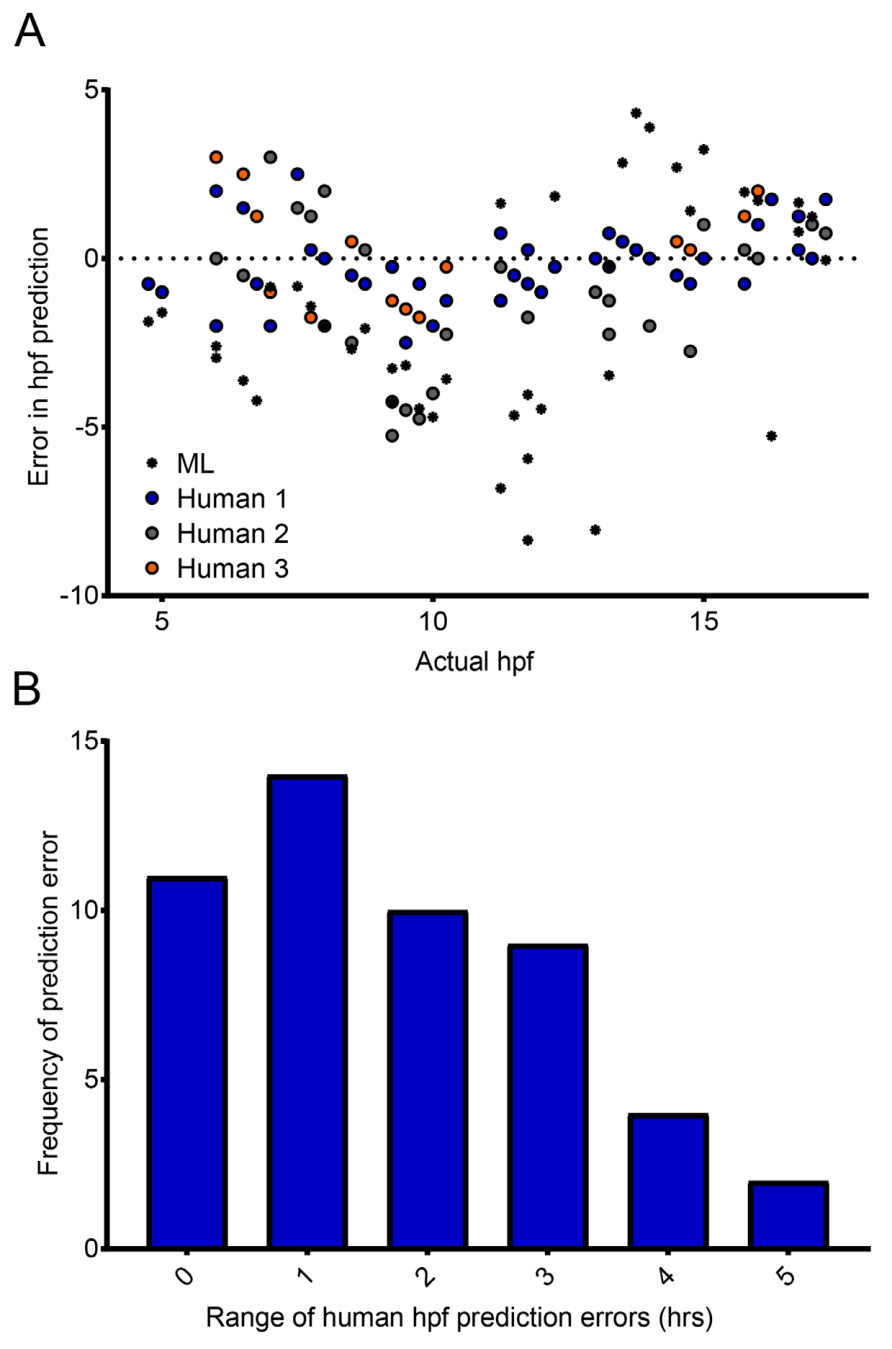
Comparison of manual and automated predictions of developmental stage. ( **A**) Machine Learning (ML) classifier- and human-predicted hpf for 42 images of zebrafish embryos ranging from 4 to 17.5 hpf – each dot represents a single prediction for a single image. ( **B**) Distribution of the range of human-predicted hpfs in ( **A**), where the range represents the difference between the maximum and minimum error at each timepoint in ( **A**).

Having shown that our classifier can make meaningful relative comparisons between the developmental speed of embryos incubated at different temperatures, we next asked how accurate our classifier is at determining the actual developmental stage of specific embryos. More specifically, could our classifier identify the actual developmental stage, in hpf, of the embryos imaged? Importantly, our classifier was trained to give the probability that a given embryo belongs to one of two classes (4.5 hpf or 17.5 hpf) with the intention of detecting developmental delays. However, we were interested to ask how it compared with manual (human) staging. Crucially, images captured and assessed by the classifier are not controlled in relation to embryo orientation. In practice, this means that in some images, the embryonic stage can be clearly seen and identified ( *e.g.* by counting the somites). In other images however, it is much more difficult, because the embryo is in an orientation in which key morphological features cannot be distinguished, or indeed the image itself is blurred. Therefore, unsurprisingly, considerable variation was observed in the manual staging between three individuals — for approximately 60% of timepoints, the maximum difference between any two human estimates was two hours or greater ( Figure 4a). The random orientation of the embryos imaged in our system frequently did not permit the counting of somites, nor clear visualization of a specific developmental landmark such at the otic vesicle. Our data therefore demonstrate the importance of having multiple people stage the same samples to reach a consensus where the images are obtained in an automated fashion. When the same images were analyzed by our classifier, even given the training limitations described above, it was able to estimate the specific hpf of embryos with a similar success rate to manual (human) staging ( Figure 4b). The errors produced by the classifier (0.0 ± 0.804; mean ± 95% confidence interval) are comparable to the errors made by humans (0.0 ± 0.239). But given the imbalance in the number of data points in each population (42 versus 126), making any kind of rigorous statistical analysis is difficult. What these data do show is that despite the classifier not having been trained to identify discrete developmental timepoints, it still fares well compared to humans, and is capable of analyzing images far more rapidly.

## Discussion

Machine learning approaches in developmental biology are not new and have become increasingly popular as our ability to generate large amounts of data has evolved ( Jones, 2019; Tarca *et al.,* 2007). The generation of ‘big data’, particularly from ‘omics’ technologies, has necessitated ever more sophisticated analysis tools, and the collection of live-imaging data is no different. Our ability to obtain thousands of images of hundreds of live biological samples means there is an increasing need for more automated methods of analysis. Moreover, automated data analysis helps to minimize the proclivity for human error and unconscious bias, a particular problem in our perception of images ( Jost & Waters, 2019).

In this work, we have developed a new machine learning classifier for quantification of temporal development of the zebrafish, a commonly used model organism, particularly in the field of developmental biology. Until now, identification of developmental delay in mutant or treated zebrafish lines has only been possible by human observation and manual staging; a methodology inherently restricted in terms of numbers of embryos that can be observed over a given time-course. Moreover, as our data have shown, there is an intrinsic subjectivity in manual staging that may render results hard to reproduce, for example, over half of the images assessed by humans in our study showed at least a 2 hpf variability between individuals, and in some cases, considerably more. Our classifier at present uses relatively simple brightfield images, and therefore accuracy could be improved by incorporating gene expression data using fluorescent transgenic reporter lines. The expression profiles of numerous key genes during zebrafish development are clearly defined both spatially and temporally, so it follows that we could improve the accuracy of our classifier by the addition of gene expression data of selected genes. These could include for example, *tbxta* ( *brachyury, T, no tail*) (germ-ring from ~5 hpf, notochord from ~10 hpf) ( Schulte-Merker *et al.,* 1992; Schulte-Merker *et al.,* 1994), *sox10* (neural crest from ~10 hpf) ( Dutton *et al.,* 2001) and *myod* (presumptive mesoderm from ~5hpf, somites from ~10hpf) ( Weinberg *et al.,* 1996). In a similar way, Pond *et al.* (2021) incorporated gene expression data from confocal microscopy images to enhance their algorithm training, using fluorescent *in situ* hybridisation techniques to profile gene expression. Although it precludes the use of fluorescent *in situ* hybridisation techniques to profile gene expression, a key advantage of our classifier system is the use of live imaging, whereby the course of developmental progression is captured, as opposed to a series of fixed images of different embryos. Moreover, another key strength of our classifier is its ability to accurately quantify temporal development from images of embryos in random orientations with absolutely no image quality control. We envisage that our classifier will be a particularly useful tool in studies where accurate quantification of developmental delay is imperative, such as for developmental toxicity testing of drugs and toxicants ( Dasgupta *et al.*, 2020; Nishimura *et al.*, 2016; Song *et al.*, 2021).

Limitations of this study include testing one 96-well plate at a given time meaning the 28.5°C and 25°C experiments were conducted on different days, and the lack of testing using a genetically perturbed/drug-treated zebrafish line. Additionally, in cases where only a small sample of embryos is to be tested (e.g. <50), it may be less labor-intensive to monitor development manually, albeit with appropriate controls to reduce subjectivity.

Other studies have used 3D imaging and OPT to enhance the ability of machine learning approaches to accurately stage and identify morphological features ( Guglielmi *et al.,* 2021; Pond *et al.,* 2021). Our classifier at present uses relatively simple 2D images, taken using a standard wide-field microscope, and its simplicity in both image acquisition and analysis makes it accessible to a wide audience. Similarly, although our classifier has been trained using WT embryos, the same pipeline could be used to analyze zebrafish embryos with aberrant morphologies, e.g. the *no tail* ( *Brachyury*) mutant ( Halpern *et al.,* 1993; Schulte-Merker *et al.,* 1994), providing the algorithm is retrained on a subset of the given mutant embryos.

Finally, while we implemented our classifier using “conventional” image analysis tools such as ilastik and FIJI, the use of deep learning in biological research is becoming ever more popular ( Hallou *et al.,* 2021). However, the application of deep learning for staging zebrafish embryos would require optimization of neural network architecture, along with a substantially larger volume of training data — this requires considerable computational time and resources.

## Conclusion

The developing zebrafish embryo is used in many different types of studies and accurate staging is essential. When comparing an experimental group of embryos with a control group, ensuring the embryos have reached the same developmental stage allows for meaningful comparisons to be made. Moreover, identification of a developmental delay in an experimental group is itself an important phenotypic observation. Our machine learning based classifier enables the unbiased assessment of thousands of images, across hundreds of embryos, with minimal time commitment. We anticipate that our classifier will be a useful tool for the zebrafish community to determine whether experimental animals (mutants, morphants, drug treated embryos) develop at the same rate as WT counterparts.

## Data Availability

All image data generated in this study is available to download from the BioImage Archive (accession number S-BIAD531) https://www.ebi.ac.uk/biostudies/bioimages/studies/S-BIAD531 Data are available under the terms of the Creative Commons Zero "No rights reserved" data waiver (CC0 1.0 Public domain dedication). Zenodo: ARRIVE 2.0 checklist for "Automated staging of zebrafish embryos using machine learning" https://doi.org/10.5281/zenodo.7198533 ( Barry, 2022a) Data are available under the terms of the Creative Commons Attribution 4.0 International license (CC-BY 4.0).

## Software availability

Source code available from: https://github.com/djpbarry/fish-quant

Archived source code available from: https://doi.org/10.5281/zenodo.7189408 ( Barry, 2022).

License: GNU v3.0
