# Supplementary material for: Automated staging of zebrafish embryos using machine learning
Source: Wellcome Open Res. 2023 Apr 26;7:275. Originally published 2022 Nov 9. [Version 3] doi: 10.12688/wellcomeopenres.18313.3 (PMC10442596; doi:10.12688/wellcomeopenres.18313.3)
Supplement: Peer review history

# Peer review: Reviewer response for version 3

**DOI:** 10.21956/wellcomeopenres.21485.r64540

**Published:** 2023-08-21

**Authors:** Christian Tischer, Arif Khan, Sebastian Gonzalez-Tirado

**Affiliations:** 1 Centre for BioImage Analysis, European Molecular Biology Laboratory, Heidelberg, Germany; 2 EMBL Heidelberg, Heidelberg, Germany

**# Overall assessment**

 In this manuscript the authors describe a computational method for staging zebrafish embryos.

 Firstly, we would like to congratulate the authors for publishing all image data on the Bioimage Archive and putting all code onto a publicly accessible github repository.

 However, in our opinion, the overall rationale does not scientifically adhere to the standards in the field. The authors use a linear regression to interpolate random forest probabilities, which are not meant to provide a linear response to the input data. It is not obvious whether the object features that are used as an input to the random forest should change linearly during zebrafish development. For example, maybe the “variance in intensity” ( https://www.ilastik.org/documentation/objects/objectfeatures) would change only in later stages while it stays constant for some duration during early development, potentially resulting in a non-linear response. The random forest classifier is adding even more nonlinearities as each decision tree is composed of several thresholds on the image features and the vote of each tree is a majority vote of all its leaves. The ilastik probabilities summarize the votes of all trees. Thus, overall, the rationale of a linear interpolation of those, by design, highly non-linear probabilities is in our view scientifically questionable. We feel that this notion is supported by the data. In our view, the data points for plate 1 in Figure 3A could be well fit by a step function with three steps where the random forest output probabilities stay relatively constant from 4 to 6 hpf and from 6 to 13 hpf and from 13 to 20 hpf, consistent with the non-linear design of the random forest that is meant to create decisions rather than a linear response.

 Along those lines, we were also wondering that why the authors chose to paint a schematic non-linear response in Figure 1E. Could you please comment?

 We would like to offer two suggestions for how to modify the approach of the authors while still keeping a large part of the overall pipeline intact:

  

1. As far as we know, the measured features can be extracted from ilastik and it would be very interesting to plot all of them against hpf and see whether some of them change linearly. If yes, those linearly responding features could be used to train a linear regression model to achieve the aim of the publication.
2. As an alternative, the authors could decide to split the developmental time, e.g. into bins of 2 hours and then train ilastik to predict into which bin the embryo belongs. That is, for 20 hours one would need to train 10 classes. We hope that this should be an acceptable effort in terms of annotations. The output of the overall pipeline would then simply be the class that is predicted by ilastik with the highest probability.

Another general comment is that for all approaches it is critical to keep the imaging conditions identical in terms of illumination intensity and exposure time, to ensure that the algorithm trained on one data set can be applied to another one. It would be important to mention this in the manuscript. In addition, it should be considered whether the images could be normalized, e.g. using intensity percentiles, before being input to the algorithms. In fact, we think it would be essential to test whether such a normalization could improve the robustness of the results, especially when staging embryos that are imaged on  different days and that were not part of the training data.

**# Manuscript**

 Page3- Text: *“The staging of  ….. well-suited”*. A reference (citation) would be helpful.

 Page3- Text: *“Jeanray et al.   ….. Shang et al. (2022)”*. Hard to comprehend. Please rephrase.

 Page3- Text: *“ … semi-automated segmentation and quantitation …. ”*.  Use quantification ?!

 Page4- Text: *“ … using simple grey level threshold …. ”*. What is meant by ‘simple’ here? What was the criterion for threshold selection? We think it is generally good to avoid qualifications such as “simple”.

 Page5- Table1: Could you please elaborate in the text why no data from plate 3 was used for training?!

 Figure 2, A: How were these features for pixel classification selected? Can you please explain the rationale why these features were used and which filter sizes were selected? In case, the authors just used all features that are available in ilastik, we think this should be mentioned like this instead of listing them individually.

 Figure 2, B: Can you please label the individual morphological operation steps?

 Figure 2,- Caption B: What kind of thresholding and what morphological operations were used?

 Figure 2, C: How were these features  selected?

 Page8- Text: *“….. image itself is blurred”*. Why are the images blurred? Could they be removed from the data set?

 Page8- Text: *“….. Test data was not subjected to any quality control, …..”*. Why not?

 Page8- Text: *“But given the imbalance   ….. analysis is difficult”*. Why was this imbalance created in the first place?

 Page8- Text: *“ ….. analyzing images far more rapidly”*. Do you have any estimation on how fast in minutes/hours/….?

 Page10- Text: *“ When comparing ….. To be made”*. Not comprehensible. Can you please rephrase?

 Page10- Text: *“ ….. unbiased assessment …..”*.  Replace with automated?

**# Software**

 In general, we feel that the software is missing documentation, especially to make the usage of it clear to non-computational users that would like to use it. For example, there is no information on how a user could start using the software. Information such as *“clone the repository, you will find the trained models inside this directory, etc”* is missing. Other basic information is also missing on describing how each command should be executed. For example, part of the first step of the pipeline is written as:

 ❯ **/run_ilastik.sh … --output_filename_format="/{nickname}_{result_type}.tiff" "input.tiff"**

 But there are no comments indicating that the user should replace the parts between angle brackets (< >) with their own input. Also, please comment about the importance of keeping the other brackets (such as in **{nickname}_{result_type}.tiff**) for the software to work properly.

 The Fiji scripts are devoid of comments in the code describing the different steps, functions, etc.

 We think that the repository should have a minimal example pointing to a dataset that could be used to run the software with the exact instructions to showcase how to run it!

 Regarding other technical issues, we found that the data was missing some information in their metadata. For example, when opening the image properties on Fiji, we could not find the value of the frame intervals (it was zero and it would be great if it was giving the hpf).

 Also, there should be information regarding the software versions used to run the analysis. We encourage this last point since we were not able to reproduce the workflow using the following time-series from the test-set:

**FishDev_WT_01_1_MMStack_A8-Site_0.ome.tiff**

 First, we noticed that the first script of the tool only outputs the pixel predictions for two time-points, which was not clear if this is how the tool is supposed to work or whether this is a bug and one should expect the pixel predictions for all time points of the dataset. Also, we could not reproduce the whole workflow since we had an error after attempting to run the object classification part of the workflow (step 3, error input/output marked below).

 ## Error output from step 3:

 ❯ /Applications/ilastik-1.4.0b27-OSX.app/Contents/ilastik-release/run_ilastik.sh --headless --project="./ObjectClassifier.ilp" --export_source="Object Probabilities" --output_format="multipage tiff"  --output_filename_format="./{nickname}_{result_type}.tiff" --raw_data="/Users/segonzal/Downloads/Zebrafish_ML_Archive/test_data/FishDev_WT_01_1/FishDev_WT_01_1_MMStack_A8-Site_0.ome.tif" --segmentation_image="/Users/segonzal/Downloads/Zebrafish_ML_Archive/test_data/FishDev_WT_01_1/FishDev_WT_01_1_MMStack_A8-Site_0.ome_Probabilities.tiff_Binary.tiff" --export_dtype="float32" --readonly="true" > error.txt

 "/Applications/ilastik-1.4.0b27-OSX.app/Contents/ilastik-release/lib/python3.7/site-packages/lazyflow/operators/ioOperators/opTiffReader.py", line 58, in setupOutputs

     assert axes[last_C_pos] == "C"

 AssertionError

Are the conclusions about the tool and its performance adequately supported by the findings presented in the article?

No

Is the rationale for developing the new software tool clearly explained?

Yes

Is the description of the software tool technically sound?

No

Are sufficient details of the code, methods and analysis (if applicable) provided to allow replication of the software development and its use by others?

Partly

Is sufficient information provided to allow interpretation of the expected output datasets and any results generated using the tool?

Yes

Reviewer Expertise:

Bioimage analysis

We confirm that we have read this submission and believe that we have an appropriate level of expertise to confirm that it is of an acceptable scientific standard, however we have significant reservations, as outlined above.

# Peer review: Reviewer response for version 2

**DOI:** 10.21956/wellcomeopenres.21251.r55612

**Published:** 2023-03-31

**Authors:** Amin Allalou

**Affiliations:** 1 Division of Visual Information and Interaction, Department of Information Technology, Image Analysis and Human-Computer Interaction, Uppsala University, Uppsala, Sweden; 2 Science for Life Laboratory BioImage Informatics Facility, Uppsala, Sweden

Responses (1) 

  AUTHOR RESPONSE16 MARCH 2023 

 Rebecca Jones 

 The Francis Crick Institute, 1 Midland Road, London, UK 

 Responses to Reviewer 2 (Amin Allalou) In this manuscript the author is describing a method for automatic detection of different development stages for the zebrafish. A robust method that can accurately classify a zebrafish into different development stages is highly relevant for many researchers working with zebrafish and hence the importance in developing methods like this is high. 

 We thank the reviewer for their positive comments.   

 The dataset used for the method is quite small and limited. It is good for proving a proof of the concept of the method but for a more general use of the method it needs to be further tested with embryos with different genetic backgrounds or treatment (as mentioned by the author in the discussion). 

 We agree with the reviewer that the data used in this study is limited, in the sense that the images used are all of wild type zebrafish embryos and all images were acquired on the same microscopy system. However, the aim of the manuscript was indeed to demonstrate a proof of concept (that developmental delay could be quantified in an automated manner). We did explore the possibility of testing our method on images of zebrafish whose development was perturbed either by some pharmacological treatment or genetic mutation, but, as far as we are aware, no such treatments or mutants are readily available. Therefore, including such additional data would constitute a substantial body of additional work and experimentation.   

**- I see your point even though more data would make the manuscript more appealing.**  

 The author should improve the overall description of the method and the data used for training. It is sometimes difficult to understand what dataset is used for training and for evaluation. Same data cannot be used in training as in the evaluation of the method, it needs to be made clear to the reader that this is not the case. 

 We thank the reviewer for drawing our attention to this and agree that the wording of the manuscript could be improved in places. We have made clarifications regarding the nature of the training and test data, detailed in the responses below.   

**- Thank you for clarifying this.**

 Page 4 Zebrafish husbandry and Live imaging Later in the paper the author is discussing 2 different datasets for 28.5 degree data (rep1 and rep2). Could the author in this section describe the difference between those to datasets. 

 We thank the reviewer for bringing this to our attention. We have revised the methods section and added a table (Table 1) to clarify exactly what data was used for training and what was used for testing. In total, we imaged three 96-well plates of embryos, two at 28.5C and one at 25.0C. A random selection of wells from the two 28.5C plates were used for training purposes, while the remainder of wells in those plates were reserved for testing. None of the training data was subsequently used for testing. The plate incubated at 25.0C was used for testing only. To be certain that none of the training data "leaked" into the test datasets, we modified the R code used to generate the plots in Figure 3 to explicitly rule out (by name) the inclusion of the wells used for training. New plots for Figure 3 have been generated with this revised R code which, as far as we can tell, are identical to those included with the original submission.   

**- Table 1 is a good addition that makes the data selection much more clear.**

 Page 4 “A total of 20 embryos were used for training, selected at random from the two”... Why only 20 embryos? 

 This was in fact a typo (the correct number, as per Table 1, is 24). But the reason for the small number is the requirement for manual annotation, which is time consuming. However, we view this as a positive - with relatively little training data, the classifier was still successful in distinguishing between two different populations of embryos.   

**- Thank you. I agree a small number of training samples that can generate a good method is positive.**

 How are these samples used in the training. Is the set divided into training, validation and test set? This is usually the procedure. 

 The manner in which the data is divided into training and test sets is outlined in Table 1.   

**- Thank you**

 “The resultant masks were then combined with the raw pixel data in an ilastik object classification pipeline (ObjectClassifier.ilp; Barry, 2022), whereby the embryos were manually classified as being either 4.5 or 17.5 hpf (Figure 2C)."Some more detail on the classification method would be useful for the reader. 

 We have now added some further details on the object classification workflow and included a link to the relevant section of the ilastik documentation ( https://www.ilastik.org/documentation/objects/objects), should the reader require further information.   

**-Thank you**

 Page 5 “…for the same 50 images, and plotted accordingly”. Where are they plotted? 

 This corresponds to the data shown in Figure 4   

 Section “Results An accuracy measure of the correct classification would be good to have. How many samples are correctly classified into the correct class. 

 We agree with the reviewer that such a metric would be useful. However, while our machine learning model is trained on the basis of placing objects into one of two different classes (4.5 or 17.5 hpf embryos), the result is essentially a regression model, as we are testing this classifier on embryos at various stages of development between 4.5 and 17.5 hpf (inclusive). So, while we could certainly include a measure of how successful our model would be in discriminating between 4.5 and 17.5 hpf embryos, this would tell us little about how successful the model is at discriminating between different rates of development, which was our primary goal.   

**- Ok, thank you for the clarification.**

 Development of embryos is slower when they are maintained at 25°C than at 28.5°C. As proof of principle, following training on 14 embryos and subsequent testing on two 96-well plates of WT embryos at 28.5°C as …” What was trained here? The author should be more clear on what was trained here. Is anything from previous training used here (where 20 embryos were used) or is it a completely new training? Was the same classes used in the training as previous? If so why was it retrained? 

 We agree with the reviewer that this section was poorly worded and likely to cause confusion. We have therefore revised this text to make clear that no new training data has been introduced at this point - the results section refers to the same datasets referred to in the methods (and now listed in Table 1).   

**- Great!**

 If all training was done on one batch from 28.5°C it should not be used as a comparison in the evaluation where the same batch is compared to data from a new batch. This is not a valid comparison. 

 We agree with the reviewer on this point and have made changes to the text (and added Table 1) to clarify what was used as training data and what was used for testing - none of the data shown in Figure 3 was used in training.   

**-OK!**

 “our classifier was trained to give the probability that a given embryo belongs to one of two classes (4.5 hpf or 17.5 hpf) with the intention of detecting developmental delays – it was not trained to predict the actual hpf of a given embryo.” It is not really clear to the reader how the author is estimating the predicted hpf. The classifier seems to be trained on only two classes 4.5hpf and 17.5hpf. The output from the classification is a probability of belonging to one of each class. From these values how is the probability based predicted hpf in Figure 3 calculated? This should be clarified. 

 We thank the reviewer for drawing our attention to this omission. We have now added text to the relevant methods section explaining how the probability values output by the object classifier are converted into predicted hpf values.   

**-Ok, thanks for the clarification. However, it is a little bit confusing when you state “it was not trained to predict the actual hpf of a given embryo”, but you provide a regression where you try to do exactly this and also plot results for this. And at the same time you are not providing any classification accuracy. You are merely using a 2 class classifier and depending on how similar they are to the different classes you estimate the hpf. Figure 3B should be a straight line if the hpf prediction was good. Now it looks more like they are closer to one class and around 12 hpf they just shift from one class to the other. And for the 25 degree this shift happens around 16hpf. This to me does not seem like a good prediction of the hpf. However, this still shows the delay in development that the paper is aiming for but the importance and need of the regression part is not clear to me.**

 “hpf of embryos with a similar success rate to manual (human) staging (Figure 4b). The errors produced by the classifier (0.0 +-} 0.804; mean +-} 95% confidence interval) are comparable to the errors made by humans (0.0 +-} 0.239).” Is both rep 1 and rep 2 data used in this calculation? Are any of these batches used in the training? Please, clarify in the text. 

 A random selection of images from both plate 1 and plate 2 were used to generate this data. We have now noticed that, although none of the images used in training were included in this data, some images from the same wells as those used for training were included – these have now been removed and the figure revised. While there are now slightly fewer data points (44), the overall conclusion remains unchanged.   

**-OK!**

 “For example, at an actual hpf of 16.0, our classifier gave predicted hpfs of 16…. embryos and embryos maintained at 25°C, respectively.” This only provides info on that the development on the 25°C is slower, as it should be. But how accurate is the 10.21 ± 0.44 really? How much slower should an embryo at 25°C be? Did the author do some manual staging to compare this number with manual estimation? 

 We agree with the reviewer that this statement of accuracy was of limited value and have now removed it from the text. Estimations of accuracy are now limited to the data comparing our machine learning classifier with manual staging as shown in Figure 4.   

**-OK**

 Figure 1E What data is used here? It looks much cleaner than the ones shown in Figure 3? If this is just an artificial plot than are all three colors needed? 

 We have now updated the panel in Figure 1E to make it clear that this plot is not real data and is intended for illustrative purposes only.   

**- Ok, great!**

 Figure 3 In Figure 3b the spread of the data points for red (WT rep 2) seems much greater than for green (WT rep 1) (this can also be seen in the confidence interval in Fig 3b). It is not clear, but if the data for “rep 1” is coming from the same dataset as was used for the training, then this pattern is an indication that the method does not generalise well to a new batch. Could the author clarify where the data is coming from and provide a plot with the data separated? In addition, some quantification of the spread of the data in rep1 vs rep 2 would be of interest to the reader. 

 All of the data presented in Figure 3 was generated on test data only, previously unseen by the classifier. With regard to the differences in data spread, we have now added some discussion of this in the main text. Slightly more training data was drawn from plate 1 than plate 2, which may explain the slightly greater spread of data points for plate 2. It should also be noted that no quality control was applied to the test data (as this is laborious and time-consuming), so images of dead embryos and/or embryos that have drifted to the edge (our outside) of the field of view will have been included - it's possible that plate 2 produced a greater quantity of these lower quality images than plate 1. But the standard error of the mean for both plates is still less than 1.0 hpf.   

**- One problem I see with the division of the data is that in your true test set (data coming from an isolated batch) you only have data that should show a difference from your data used in the training. No real isolated control data. To make the results more clear an isolated test set for 28 degrees and 25 degrees would be a much stronger evidence of the method performance. What if the method is doing some overfit to the two training batches, how do you know the performance on the test set is accurate when you don’t have a control group (28 degree)?**

 (3b) From the graph there seems to be a cluster of blue (25 degree) with actual hpf 4-10 being classified as approx 15 hpf. A comment on this pattern would be useful to the reader since the error is quite large. 

 The majority of these datapoints correspond to the same two wells on that particular plate and it appears that segmentation error is to blame for the misclassification, as illustrated in the example below – in both cases, the embryos in the early stages of development were consistently over-segmented, which likely resulted in their misclassification.   

**-ok, If this is the case then an additional plot with those wells removed (or marked in some way  in your current plot) would clearly show the reader that the errors are coming from those wells.**

****

Figure 4. “Machine Learning (ML) classifier- and human-predicted hpf for 50 images of zebrafish” Are the data taken from the same batch as was used for training? No, as explained above, we have now made absolutely sure that none of the random images used for the human-ML comparison were drawn from the training data. 

**- Ok!**

Are the conclusions about the tool and its performance adequately supported by the findings presented in the article?

Partly

Is the rationale for developing the new software tool clearly explained?

Yes

Is the description of the software tool technically sound?

Yes

Are sufficient details of the code, methods and analysis (if applicable) provided to allow replication of the software development and its use by others?

Yes

Is sufficient information provided to allow interpretation of the expected output datasets and any results generated using the tool?

Partly

Reviewer Expertise:

Quantitative Microscopy, Image analysis, Machine learning, zebrafish

I confirm that I have read this submission and believe that I have an appropriate level of expertise to confirm that it is of an acceptable scientific standard, however I have significant reservations, as outlined above.

# Response

**Published:** 2023-04-07

**Authors:** Rebecca Jones

**Affiliations:** Princeton University, USA

Ok, thanks for the clarification.

 However, it is a little bit confusing when you state “ *it was not trained to predict the actual hpf of a given embryo*”, but you provide a regression where you try to do exactly this and also plot results for this. And at the same time you are not providing any classification accuracy. You are merely using a 2 class classifier and depending on how similar they are to the different classes you estimate the hpf. Figure 3B should be a straight line if the hpf prediction was good. Now it looks more like they are closer to one class and around 12 hpf they just shift from one class to the other. And for the 25 degree this shift happens around 16hpf. This to me does not seem like a good prediction of the hpf. However, this still shows the delay in development that the paper is aiming for but the importance and need of the regression part is not clear to me.

 We agree with the reviewer's point here that, ideally, a plot of predicted hpf versus actual hpf should result in a straight line. In our case, we have curves that could more accurately be described as s-curves, due to the nature of the model we trained, as outlined above by the reviewer. While such curves are certainly not ideal for accurately predicting the hpf of individual embryos, they are completely adequate for the identification of developmental delay, which was our primary goal.

 It is certainly possible that we could improve our model by including additional training classes (perhaps resulting in a predicted versus actual hpf plot more closely resembling a straight line), but as previously stated, this would require a substantial volume of additional work to manually annotate new training data. We did actually consider presenting our data in a slightly different manner, which would have involved labelling the y axis in Figure 3B as "Probability of embryo being 17.5 hpf", but felt that this was perhaps a little abstract and may be difficult for biologists to interpret. But we also felt that "predicted hpf" wasn't quite accurate, for the reasons the reviewer has outlined, which is why we settled on (the slightly cumbersome!) "probability based predicted hpf".

 We also agree that the statement " *it was not trained to predict the actual hpf of a given embryo*" is confusing and have removed this statement from the text.    

 One problem I see with the division of the data is that in your true test set (data coming from an isolated batch) you only have data that should show a difference from your data used in the training. No real isolated control data. To make the results more clear an isolated test set for 28 degrees and 25 degrees would be a much stronger evidence of the method performance. What if the method is doing some overfit to the two training batches, how do you know the performance on the test set is accurate when you don’t have a control group (28 degree)? We take the reviewers point about the 25C test data being completely isolated from the training data, whereas the 28.5C test data is drawn from the same plates as training data. However, in our opinion, over-fitting to training data was more likely had we used only a single 28.5C plate for training. We chose to use training data from both 28.5C plates for this reason.

 ok, If this is the case then an additional plot with those wells removed (or marked in some way in your current plot) would clearly show the reader that the errors are coming from those wells. We must respectfully disagree with the reviewer on this point. We believe that one of the major strengths of our approach is the lack of manual curation we have performed on the data - the method is 100% automated. While we accept that this will sometimes result in errors for individual images or wells, for a sufficiently large population of embryos (such as a 96 well plate), these errors are not significant, as is evidenced by our ability to detect developmental delay between two different populations.

 Removing erroneous datapoints based on arbitrary criteria would not only be an extremely time-consuming endeavour (requiring the manual assessment of thousands of images), we believe it would also significantly weaken our argument that no manual intervention is required in our pipeline.

# Peer review: Reviewer response for version 2

**DOI:** 10.21956/wellcomeopenres.21251.r55613

**Published:** 2023-03-30

**Authors:** Steffen Scholpp

**Affiliations:** 1 Living Systems Institute, School of Biosciences, College of Life and Environmental Sciences, University of Exeter, Exeter, UK

The amendments are sufficient, and therefore, I suggest the manuscript for indexing.

Are the conclusions about the tool and its performance adequately supported by the findings presented in the article?

Yes

Is the rationale for developing the new software tool clearly explained?

Yes

Is the description of the software tool technically sound?

Yes

Are sufficient details of the code, methods and analysis (if applicable) provided to allow replication of the software development and its use by others?

Partly

Is sufficient information provided to allow interpretation of the expected output datasets and any results generated using the tool?

Partly

Reviewer Expertise:

Morphogen trafficking in zebrafish

I confirm that I have read this submission and believe that I have an appropriate level of expertise to confirm that it is of an acceptable scientific standard.

# Peer review: Reviewer response for version 1

**DOI:** 10.21956/wellcomeopenres.20298.r53876

**Published:** 2023-01-26

**Authors:** Amin Allalou

**Affiliations:** 1 Division of Visual Information and Interaction, Department of Information Technology, Image Analysis and Human-Computer Interaction, Uppsala University, Uppsala, Sweden; 2 Science for Life Laboratory BioImage Informatics Facility, Uppsala, Sweden

In this manuscript the author is describing a method for automatic detection of different development stages for the zebrafish. A robust method that can accurately classify a zebrafish into different development stages is highly relevant for many researchers working with zebrafish and hence the importance in developing methods like this is high.

 The dataset used for the method is quite small and limited. It is good for proving a proof of the concept of the method but for a more general use of the method it needs to be further tested with embryos with different genetic backgrounds or treatment (as mentioned by the author in the discussion).

 The author should improve the overall description of the method and the data used for training. It is sometimes difficult to understand what dataset is used for training and for evaluation. Same data cannot be used in training as in the evaluation of the method, it needs to be made clear to the reader that this is not the case.

**Page 4** **Zebrafish husbandry** **and** **Live imaging**

- Later in the paper the author is discussing 2 different datasets for 28.5 degree data (rep1 and rep2). Could the author in this section describe the difference between those to datasets.

**Page 4**

- *“A total of 20 embryos were used for training, selected at random from the two”...*
  - Why only 20 embryos?
  - How are these samples used in the training. Is the set divided into training, validation and test set? This is usually the procedure.
- *“The resultant masks were then combined with the raw pixel data in an ilastik object classification pipeline (ObjectClassifier.ilp; Barry, 2022), whereby the embryos were manually classified as being either 4.5 or 17.5 hpf (Figure 2C)."*
   
  - Some more detail on the classification method would be useful for the reader.

**Page 5**

- *“…for the same 50 images, and plotted accordingly”.*
   
  - Where are they plotted?

**Section “Results **

- An accuracy measure of the correct classification would be good to have. How many samples are correctly classified into the correct class. 
- *“Development of embryos is slower when they are maintained at 25°C than at 28.5°C. As proof of principle, following training on 14 embryos and subsequent testing on two 96-well plates of WT embryos at 28.5°C as …”*
   
  - What was trained here? The author should be more clear on what was trained here. Is anything from previous training used here (where 20 embryos were used) or is it a completely new training? Was the same classes used in the training as previous? If so why was it retrained?
  - If all training was done on one batch from 28.5°C it should not be used as a comparison in the evaluation where the same batch is compared to data from a new batch. This is not a valid comparison.
- *“our classifier was trained to give the probability that a given embryo belongs to one of two classes (4.5 hpf or 17.5 hpf) with the intention of detecting developmental delays – it was not trained to predict the actual hpf of a given embryo.”*
   
  - It is not really clear to the reader how the author is estimating the predicted hpf. The classifier seems to be trained on only two classes 4.5hpf and 17.5hpf. The output from the classification is a probability of belonging to one of each class. From these values how is the probability based predicted hpf in Figure 3 calcualted? This should be clarified.
- *“hpf of embryos with a similar success rate to manual (human) staging (Figure 4b). The errors produced by the classifier (0.0 +-} 0.804; mean +-} 95% confidence interval) are comparable to the errors made by humans (0.0 +-} 0.239).”*
   
  - Is both rep 1 and rep 2 data used in this calculation? Are any of these batches used in the training? Please, clarify in the text.
- “ *For example, at an actual hpf of 16.0, our classifier gave predicted hpfs of 16…. embryos and embryos maintained at 25°C, respectively.”*
   
  - *This only provides info on that the development on the 25* *°C* *is slower, as it should be. But how accurate is the 10.21 ± 0.44 really? How much slower should an embryo at 25* *°C* *be? Did the author do some manual staging to compare this number with manual estimation?*

**Figure 1E**

- What data is used here? It looks much cleaner than the ones shown in Figure 3? If this is just an artificial plot than are all three colors needed?

**Figure 3**

- In Figure 3b the spread of the data points for red (WT rep 2) seems much greater than for green (WT rep 1) (this can also be seen in the confidence interval in Fig 3b). It is not clear, but if the data for *“rep 1”* is coming from the same dataset as was used for the training, then this pattern is an indication that the method does not generalise well to a new batch. Could the author clarify where the data is coming from and provide a plot with the data separated? In addition, some quantification of the spread of the data in rep1 vs rep 2 would be of interest to the reader.
- (3b) From the graph there seems to be a cluster of blue (25 degree) with actual hpf 4-10 being classified as approx 15 hpf. A comment on this pattern would be useful to the reader since the error is quite large.

**Figure 4. **

- *“Machine Learning (ML) classifier- and human-predicted hpf for 50 images of zebrafish”*
   
  - Are the data taken from the same batch as was used for training?

Are the conclusions about the tool and its performance adequately supported by the findings presented in the article?

Partly

Is the rationale for developing the new software tool clearly explained?

Yes

Is the description of the software tool technically sound?

Yes

Are sufficient details of the code, methods and analysis (if applicable) provided to allow replication of the software development and its use by others?

Yes

Is sufficient information provided to allow interpretation of the expected output datasets and any results generated using the tool?

Partly

Reviewer Expertise:

Quantitative Microscopy, Image analysis, Machine learning, zebrafish

I confirm that I have read this submission and believe that I have an appropriate level of expertise to confirm that it is of an acceptable scientific standard, however I have significant reservations, as outlined above.

# Response

**Published:** 2023-03-06

**Authors:** Rebecca Jones

**Affiliations:** Princeton University, USA

**Responses to Reviewer 2 (Amin Allalou)** *In this manuscript the author is describing a method for automatic detection of different development stages for the zebrafish. A robust method that can accurately classify a zebrafish into different development stages is highly relevant for many researchers working with zebrafish and hence the importance in developing methods like this is high.*

 We thank the reviewer for their positive comments.  

*The dataset used for the method is quite small and limited. It is good for proving a proof of the concept of the method but for a more general use of the method it needs to be further tested with embryos with different genetic backgrounds or treatment (as mentioned by the author in the discussion).*

 We agree with the reviewer that the data used in this study is limited, in the sense that the images used are all of wild type zebrafish embryos and all images were acquired on the same microscopy system. However, the aim of the manuscript was indeed to demonstrate a proof of concept (that developmental delay could be quantified in an automated manner). We did explore the possibility of testing our method on images of zebrafish whose development was perturbed either by some pharmacological treatment or genetic mutation, but, as far as we are aware, no such treatments or mutants are readily available. Therefore, including such additional data would constitute a substantial body of additional work and experimentation.  

*The author should improve the overall description of the method and the data used for training. It is sometimes difficult to understand what dataset is used for training and for evaluation. Same data cannot be used in training as in the evaluation of the method, it needs to be made clear to the reader that this is not the case.*

 We thank the reviewer for drawing our attention to this and agree that the wording of the manuscript could be improved in places. We have made clarifications regarding the nature of the training and test data, detailed in the responses below.  

*Page 4 Zebrafish husbandry and Live imaging* *Later in the paper the author is discussing 2 different datasets for 28.5 degree data (rep1 and rep2). Could the author in this section describe the difference between those to datasets.*

 We thank the reviewer for bringing this to our attention. We have revised the methods section and added a table (Table 1) to clarify exactly what data was used for training and what was used for testing. In total, we imaged three 96-well plates of embryos, two at 28.5C and one at 25.0C. A random selection of wells from the two 28.5C plates were used for training purposes, while the remainder of wells in those plates were reserved for testing. None of the training data was subsequently used for testing. The plate incubated at 25.0C was used for testing only. To be certain that none of the training data "leaked" into the test datasets, we modified the R code used to generate the plots in Figure 3 to explicitly rule out (by name) the inclusion of the wells used for training. New plots for Figure 3 have been generated with this revised R code which, as far as we can tell, are identical to those included with the original submission.  

*Page 4* *“A total of 20 embryos were used for training, selected at random from the two”...* *Why only 20 embryos?*

 This was in fact a typo (the correct number, as per Table 1, is 24). But the reason for the small number is the requirement for manual annotation, which is time consuming. However, we view this as a positive - with relatively little training data, the classifier was still successful in distinguishing between two different populations of embryos.  

*How are these samples used in the training. Is the set divided into training, validation and test set? This is usually the procedure.*

 The manner in which the data is divided into training and test sets is outlined in Table 1.  

*“The resultant masks were then combined with the raw pixel data in an ilastik object classification pipeline (ObjectClassifier.ilp; Barry, 2022), whereby the embryos were manually classified as being either 4.5 or 17.5 hpf (Figure 2C)."* *Some more detail on the classification method would be useful for the reader.*

 We have now added some further details on the object classification workflow and included a link to the relevant section of the ilastik documentation (https://www.ilastik.org/documentation/objects/objects), should the reader require further information.  

*Page 5* *“…for the same 50 images, and plotted accordingly”.* *Where are they plotted?*

 This corresponds to the data shown in Figure 4  

*Section “Results* *An accuracy measure of the correct classification would be good to have. How many* *samples are correctly classified into the correct class.*

 We agree with the reviewer that such a metric would be useful. However, while our machine learning model is trained on the basis of placing objects into one of two different classes (4.5 or 17.5 hpf embryos), the result is essentially a regression model, as we are testing this classifier on embryos at various stages of development between 4.5 and 17.5 hpf (inclusive). So, while we could certainly include a measure of how successful our model would be in discriminating between 4.5 and 17.5 hpf embryos, this would tell us little about how successful the model is at discriminating between different rates of development, which was our primary goal.  

*Development of embryos is slower when they are maintained at 25°C than at 28.5°C. As proof of principle, following training on 14 embryos and subsequent testing on two 96-well plates of WT embryos at 28.5°C as …”* *What was trained here? The author should be more clear on what was trained here. Is anything from previous training used here (where 20 embryos were used) or is it a completely new training? Was the same classes used in the training as previous? If so why was it retrained?*

 We agree with the reviewer that this section was poorly worded and likely to cause confusion. We have therefore revised this text to make clear that no new training data has been introduced at this point - the results section refers to the same datasets referred to in the methods (and now listed in Table 1).  

*If all training was done on one batch from 28.5°C it should not be used as a comparison in the evaluation where the same batch is compared to data from a new batch. This is not a valid comparison.*

 We agree with the reviewer on this point and have made changes to the text (and added Table 1) to clarify what was used as training data and what was used for testing - none of the data shown in Figure 3 was used in training.  

 “ *our classifier was trained to give the probability that a given embryo belongs to one of two classes (4.5 hpf or 17.5 hpf) with the intention of detecting developmental delays – it was not trained to predict the actual hpf of a given embryo.”* *It is not really clear to the reader how the author is estimating the predicted hpf. The classifier seems to be trained on only two classes 4.5hpf and 17.5hpf. The output from the classification is a probability of belonging to one of each class. From these values how is the probability based predicted hpf in Figure 3 calculated? This should be clarified.*

 We thank the reviewer for drawing our attention to this omission. We have now added text to the relevant methods section explaining how the probability values output by the object classifier are converted into predicted hpf values.  

*“hpf of embryos with a similar success rate to manual (human) staging (Figure 4b). The errors produced by the classifier (0.0 +-} 0.804; mean +-} 95% confidence interval) are comparable to the errors made by humans (0.0 +-} 0.239).”* *Is both rep 1 and rep 2 data used in this calculation? Are any of these batches used in the training? Please, clarify in the text.*

 A random selection of images from both plate 1 and plate 2 were used to generate this data. We have now noticed that, although none of the images used in training were included in this data, some images from the same wells as those used for training were included – these have now been removed and the figure revised. While there are now slightly fewer data points (44), the overall conclusion remains unchanged.  

*“For example, at an actual hpf of 16.0, our classifier gave predicted hpfs of 16…. embryos and embryos maintained at 25°C, respectively.”* *This only provides info on that the development on the 25°C is slower, as it should be. But how accurate is the 10.21 ± 0.44 really? How much slower should an embryo at 25°C be? Did the author do some manual staging to compare this number with manual estimation?*

 We agree with the reviewer that this statement of accuracy was of limited value and have now removed it from the text. Estimations of accuracy are now limited to the data comparing our machine learning classifier with manual staging as shown in Figure 4.  

*Figure 1E* *What data is used here? It looks much cleaner than the ones shown in Figure 3? If this is just an artificial plot than are all three colors needed?*

 We have now updated the panel in Figure 1E to make it clear that this plot is not real data and is intended for illustrative purposes only.  

*Figure 3* *In Figure 3b the spread of the data points for red (WT rep 2) seems much greater than for green (WT rep 1) (this can also be seen in the confidence interval in Fig 3b). It is not clear, but if the data for “rep 1” is coming from the same dataset as was used for the training, then this pattern is an indication that the method does not generalise well to a new batch. Could the author clarify where the data is coming from and provide a plot with the data separated? In addition, some quantification of the spread of the data in rep1 vs rep 2 would be of interest to the reader.*

 All of the data presented in Figure 3 was generated on test data only, previously unseen by the classifier. With regard to the differences in data spread, we have now added some discussion of this in the main text. Slightly more training data was drawn from plate 1 than plate 2, which may explain the slightly greater spread of data points for plate 2. It should also be noted that no quality control was applied to the test data (as this is laborious and time-consuming), so images of dead embryos and/or embryos that have drifted to the edge (our outside) of the field of view will have been included - it's possible that plate 2 produced a greater quantity of these lower quality images than plate 1. But the standard error of the mean for both plates is still less than 1.0 hpf.  

*(3b) From the graph there seems to be a cluster of blue (25 degree) with actual hpf 4-10 being classified as approx 15 hpf. A comment on this pattern would be useful to the reader since the error is quite large.*

 The majority of these datapoints correspond to the same two wells on that particular plate and it appears that segmentation error is to blame for the misclassification, as illustrated in the example below – in both cases, the embryos in the early stages of development were consistently over-segmented, which likely resulted in their misclassification.  

*Figure 4.* *“Machine Learning (ML) classifier- and human-predicted hpf for 50 images of zebrafish”* *Are the data taken from the same batch as was used for training?* No, as explained above, we have now made absolutely sure that none of the random images used for the human-ML comparison were drawn from the training data.

# Peer review: Reviewer response for version 1

**DOI:** 10.21956/wellcomeopenres.20298.r53245

**Published:** 2022-11-25

**Authors:** Steffen Scholpp

**Affiliations:** 1 Living Systems Institute, School of Biosciences, College of Life and Environmental Sciences, University of Exeter, Exeter, UK

The authors describe an automated method to stage zebrafish embryos. Using a machine learning approach, the authors developed a way to determine the stages of embryos without dechorionation from 4hpf – 18hpf automatically. Then, the authors use two different temperatures to evaluate the method's efficiency: staging embryos at 25°C and 28.5°C and comparing the results to manually staged embryos. The software seems to be less accurate than staging by researchers. However, the differences are minor and can be neglected. The obvious advantage is the possibility of staging a large number of embryos. 

 In my opinion, this is a valuable method to analyse many embryos in a short time. Moreover, the technique seems to cope reasonably well with specific challenges, such as the orientation of the individual embryos or the chorion.

 Personally, I would like to see more ways in which the method is challenged. For example, it would be interesting to see how the algorithm copes with higher temperatures, i.e. 33°C or by mutations lacking fundamental body parts, such as in the headless mutant or the no tail mutant. How would the staging work in these embryos? These experiments are not strictly required for this manuscript but would provide additional insight into how the technology works.

 The method was tested only for several stages (4 – 18hpf). Can that be extended to older stages, or does the twitching of the embryos after 20hpf affect the automated staging? Furthermore, is pigmentation an obstacle? Finally, does the method also work with fixed embryos? These aspects should be discussed.

 Finally, the advantage of the machine learning approach was not immediately apparent. For example, does the algorithm improve after staging more embryos? The authors should clarify how many iterations have been performed and if the authors predict a further improvement in the future.

 In general, this is a handy method for large chemical or genetic screens. However, with a smaller sample size, such a method seems too labour-intensive, and the specialised equipment, such as cameras and well plates, could be troublesome. Therefore, it would be interesting to read for which applications this technology would be beneficial. Similarly, it would help the readers to find a critical discussion on the limitations of this technology.

 Minor comments:

 The different colours in the plot in Fig. 1E are not explained and should refer to the explanation in Fig. 3.

Are the conclusions about the tool and its performance adequately supported by the findings presented in the article?

Yes

Is the rationale for developing the new software tool clearly explained?

Yes

Is the description of the software tool technically sound?

Yes

Are sufficient details of the code, methods and analysis (if applicable) provided to allow replication of the software development and its use by others?

Partly

Is sufficient information provided to allow interpretation of the expected output datasets and any results generated using the tool?

Partly

Reviewer Expertise:

Morphogen trafficking in zebrafish

I confirm that I have read this submission and believe that I have an appropriate level of expertise to confirm that it is of an acceptable scientific standard, however I have significant reservations, as outlined above.

# Response

**Published:** 2023-03-06

**Authors:** Rebecca Jones

**Affiliations:** Princeton University, USA

**Responses to Reviewer 1 (Stefen Schlopp)** *The authors describe an automated method to stage zebrafish embryos. Using a machine learning approach, the authors developed a way to determine the stages of embryos without dechorionation from 4hpf – 18hpf automatically. Then, the authors use two different temperatures to evaluate the method's efficiency: staging embryos at 25°C and 28.5°C and comparing the results to manually staged embryos. The software seems to be less accurate than staging by researchers. However, the differences are minor and can be neglected. The obvious advantage is the possibility of staging a large number of embryos.* *In my opinion, this is a valuable method to analyse many embryos in a short time. Moreover, the technique seems to cope reasonably well with specific challenges, such as the orientation of the individual embryos or the chorion.*

 We thank the reviewer for taking the time to read and comment on our manuscript, and for the supportive and helpful comments.  

*Personally, I would like to see more ways in which the method is challenged. For example, it would be interesting to see how the algorithm copes with higher temperatures, i.e. 33°C or by mutations lacking fundamental body parts, such as in the headless mutant or the no tail mutant. How would the staging work in these embryos? These experiments are not strictly required for this manuscript but would provide additional insight into how the technology works*.

 We agree with the reviewer that it would be interesting to see how the algorithm copes with higher temperatures, and indeed we did try this at 32°C, however the embryos did not develop well and the majority died within a few hours of starting the experiment. In terms of testing the algorithm using mutants with a different morphology to WT (e.g. the headless mutant as suggested), our classifier was only designed to detect normal WT development, and whilst the same analysis pipeline could be used, the algorithm would need to be retrained on the ‘new’ morphology. We have added an additional comment in our manuscript to reflect this, and we thank the reviewer for bringing it to our attention.  

*The method was tested only for several stages (4 – 18hpf). Can that be extended to older stages, or does the twitching of the embryos after 20hpf affect the automated staging? Furthermore, is pigmentation an obstacle? Finally, does the method also work with fixed embryos? These aspects should be discussed.*

 The reviewer asks whether the method could be extended to older stages, and this is indeed possible. The end point of the experiment (17.5 hpf) was sufficient for proof-of-concept, and there was therefore no reason for us to train the classifier beyond this stage. The end point could easily be extended, and the emerging pigmentation of the embryos as they develop would not pose a problem. Similarly, twitching of the embryos would not affect the outcome. This is because a unique advantage of our classifier is the complete absence of quality control; it is able to accurately quantify developmental temporal trajectory regardless of the orientation of the embryos. We have included a further sentence in our manuscript reflecting this, and we thank the reviewer for the helpful input. In respect of fixed embryos, this is not something we tried, and due to differences in opacity of live vs fixed embryos, we believe that the classifier may need retraining in the same way as described above.  

*Finally, the advantage of the machine learning approach was not immediately apparent. For example, does the algorithm improve after staging more embryos? The authors should clarify how many iterations have been performed and if the authors predict a further improvement in the future.*

 The reviewer has raised an interesting point regarding whether the algorithm improves after staging more embryos, and we have included some additional text in our manuscript to clarify the number of training iterations that were performed. Theoretically, the machine-learning based classifier could improve if we trained it on more WT embryos, however it is also possible to over-train the classifier, resulting in its abilities becoming too specific to the training sets and less generalizable over a wider range of samples. There is consequently a training ‘sweet-spot’, and less can be more. Deep-learning is a way to potentially address this, but discussion of this falls outside the scope of this current paper.  

*In general, this is a handy method for large chemical or genetic screens. However, with a smaller sample size, such a method seems too labour-intensive, and the specialised equipment, such as cameras and well plates, could be troublesome. Therefore, it would be interesting to read for which applications this technology would be beneficial. Similarly, it would help the readers to find a critical discussion on the limitations of this technology.*

 The reviewer comments that our method seems labor-intensive for smaller sample sizes. This is a valid point, and we have added text in the discussion section of the manuscript regarding the limitations of our classifier, which we trust addresses this issue.  

*The different colours in the plot in Fig. 1E are not explained and should refer to the explanation in Fig. 3.*

 Figure 1E has now been replaced with a simple schematic to show how the technology works; the figure legend has been updated accordingly.
